# Representation and Re-Presentation in Litigation Science

**DOI:** 10.1289/ehp.9976

**Published:** 2007-11-07

**Authors:** Sheila Jasanoff

**Affiliations:** Harvard University, Cambridge, Massachusetts, USA

**Keywords:** admissibility, evidence, expert witness, forensic science, litigation science, science studies, visualization

## Abstract

Federal appellate courts have devised several criteria to help judges distinguish between reliable and unreliable scientific evidence. The best known are the U.S. Supreme Court’s criteria offered in 1993 in *Daubert v. Merrell Dow Pharmaceuticals, Inc*. This article focuses on another criterion, offered by the Ninth Circuit Court of Appeals, that instructs judges to assign lower credibility to “litigation science” than to science generated before litigation. In this article I argue that the criterion-based approach to judicial screening of scientific evidence is deeply flawed. That approach buys into the faulty premise that there are external criteria, lying outside the legal process, by which judges can distinguish between good and bad science. It erroneously assumes that judges can ascertain the appropriate criteria and objectively apply them to challenged evidence before litigation unfolds, and before methodological disputes are sorted out during that process. Judicial screening does not take into account the dynamics of litigation itself, including gaming by the parties and framing by judges, as constitutive factors in the production and representation of knowledge. What is admitted through judicial screening, in other words, is not precisely what a jury would see anyway. Courts are sites of repeated re-representations of scientific knowledge. In sum, the screening approach fails to take account of the wealth of existing scholarship on the production and validation of scientific facts. An unreflective application of that approach thus puts courts at risk of relying upon a “junk science” of the nature of scientific knowledge.

## Science as Argument

Late one evening, I phone my son and end up talking to his wife. My son, a neuroscientist, is in his laboratory that night, trying to finish a grant application due the next day. He might have to stay there all night, my daughter-in-law resignedly observes. What he has to do is time-consuming. He is reformatting the visual representations of his data, making them more aesthetic, he hopes, and also more convincing to the funding body and its referees. Creating the right sorts of displays is not “doing science” as people conventionally think of it. Visualization becomes an issue only after the experiments are completed and the data already collected. Yet unless the results can be shown to others in convincing form, it would be almost as if the underlying observations had never been made. Good representations are essential if a scientist wishes to communicate findings beyond the laboratory—to persuade colleagues, attract sponsors, inform policy-makers, convince juries, or inspire students.

Another day my teaching assistant tells me that she has been having a frustrating time with her work. She is modeling the transport of atmospheric mercury, and she is trying to represent what she knows about airborne concentrations of the pollutant. She has been producing colored maps, using rainbow effects to shade from one concentration region to another. Her supervisor thinks this strategy is not visually intelligible enough. He urges her to use five colors, with clear boundaries and no shading. He is convinced that this technique will display the information in more understandable terms—not only to fellow scientists, but also to regulators who may eventually set standards based on the work performed in their laboratory. My assistant says it is very difficult to change from one coloring system to another, but she is trying hard.

These are routine moments in the lives of working scientists, hardly worth recording, one may think. But from the standpoint of the law, they illustrate critically important features of scientific practice and process. First, these episodes demonstrate the crucial role of representation, more particularly, visual representation, in the making of scientific arguments. Second, they display science as a form of communicative, persuasive, even argumentative activity, very similar in these respects to the law. Third, they illustrate the contingency, or local specificity, of some of the choices scientists make in producing representations of natural phenomena. There is no predetermined, single right way to make visual images of the workings of the rat brain or the movements of mercury in the atmosphere. Fourth, and finally, stories such as these make it clear that science done in laboratories has consequences in the outside world, affecting social choices such as flows of funds and formulations of policy; scientists, even in the so-called ivory towers of major research universities, are self-consciously aware of the strategic dimensions of their work.

That science progresses with more than a passing nod toward its relations with society is nothing new, and among social institutions the law has long attracted scientists’ particular attention. For more than 200 years, a sizable component of scientific activity has been dedicated to meeting the needs of the legal system in varied ways (Clark and Crawford 1994; Golan 2004). Scientific information provides justification for governmental decisions in many domains, including health, safety, and environmental regulation, economic and educational policy, and national security and defense. Equally, scientific data are required from parties seeking a wide range of governmental benefits, from approval of new products to patents on inventions. Not least, scientific evidence has become a virtual necessity in conducting both civil and criminal legal proceedings; the late twentieth century brought an upsurge in the use of forensic science as it did in expert witnessing. Three widely discussed U.S. Supreme Court decisions of the 1990s, beginning with the landmark case of *Daubert v. Merrell Dow Pharmaceuticals, Inc.* 1993, and followed by *General Electric Co. v. Joiner* 1997 and *Kumho Tire Co. v. Carmichael* 1999, signaled the legal system’s awareness of its increasing entanglement with science and the resulting need to rearticulate the criteria for admitting expert evidence into the courtroom.

*Daubert* and its progeny mark, in a sense, a low-water point in judicial self-esteem concerning the capacity of the legal process to generate or properly assess scientific knowledge. Doubt is apparent both in *Daubert*’s injunction to trial judges to become proactive gatekeepers against unreliable or irrelevant evidence—thereby taking important aspects of fact-finding away from lay juries (Berger 2001)—and in the Court’s admonition that judges should think like scientists in evaluating admissibility. Judges, in this view, should serve as inscription (or, more accurately, rein-scription) devices, automatically writing scientists’ standards of reliability and validity into their assessments of the evidence (Jasanoff 2005). This functional rearrangement of trial choreography not only raised the bar against testimony offered by civil plaintiffs, the parties most likely to be disadvantaged by *Daubert* hearings, but also demoted juries to a subordinate role in fact-finding. In *Daubert’*s epistemological framework, scientists establish the criteria for what counts as science, and judges are charged with importing these into admissibility decisions; only after claims pass through the double screen of judges “thinking like scientists” are juries entitled to hear testifying experts and weigh their respective credibility.

On rehearing *Daubert*, the Ninth Circuit Court of Appeals continued the trend toward institutional self-abnegation by questioning the reliability of litigation-generated science—that is, science conducted solely in response to issues raised by a lawsuit. At issue in both *Daubert* cases was the previously unresearched question whether the drug Bendectin ingested during pregnancy causes birth defects. Judge Alex Kozinski in effect denied the power of litigation to generate scientific knowledge untainted by the interests of the parties [but see Boden and Ozonoff (2008)]. Instead, he wrote that testimony “based directly on legitimate, preexisting research unrelated to the litigation provides the most persuasive basis for concluding” that an expert’s opinions are “derived by the scientific method” (*Daubert v. Merrell Dow Pharmaceuticals, Inc.* 1995). Kozinski, like Justice Harry Blackmun writing for the Supreme Court’s majority in *Daubert*, thereby posited the existence of two types of scientific knowledge: on one side, a domain of pure, unbiased, prelitigation science, characterized by the use of well-recognized and accredited scientific methods, in which such processes as peer review, testing, and replication ensure reliability; on the other, a domain of impure, party-driven, potentially biased “litigation science,” in which partisan interests and absence of replication or review by peers undermines the testifying expert’s claims to reliability.

Legal analysts and scholars trained in science and technology studies (STS) have rightly criticized the characterizations of both law and science offered by both *Daubert* opinions. Students of jury behavior, for example, question *Daubert*’s underlying premise of lay incompetence (Vidmar 1995), which provided much of the justification for a threshold screening by judges to keep “junk science” out of the courtroom (Huber 1991). STS analysts, for their part, note that the *Daubert* decisions rest on ideal-typical assumptions about science and the scientific method that are not borne out by observed scientific activity. Most important for the present discussion, the science needed to resolve legal disputes very often does not preexist the controversy (Jasanoff 1995), but rather is contingently constructed to answer case-specific questions that might never have arisen through pure or paradigm-generated science, or not in comparable form. The reasons for such nonexistence are many: novelty of the issues, regulatory grandfathering, low scientific interest, cost constraints, simple ignorance. The courtroom thus becomes not the first but the only forum in which competing claims of expertise and credibility can be sorted out (Bal 2005). It is not surprising, then, that rules of method that courts have claimed to borrow from science have been constructed within the processes of adjudication, as courts articulate and then transmit such rules from one jurisdiction to another. For example, the inclination by post-*Daubert* federal courts to favor epidemiologic over other forms of evidence, on the ground that epidemiology is scientifically preferred, turns out on close analysis of the Bendectin cases to be just such a judicial construct (Edmond and Mercer 2000).

Despite all criticism, however, *Daubert* remains the law of the land in the federal courts, and its narrow holding—that the Federal Rules of Evidence control admissibility rather than the 1923 decision in *Frye v. United States*—remains unchallenged. Most of *Daubert*’s implementation problems frequently arise from two other sources, both rooted in judicial constructions of how science works. The first is the rigid and formulaic application of the criteria of scientific validity announced by Justice Blackmun as merely exemplary: “Many factors will bear on the inquiry, and we do not presume to set out a definitive checklist or test” (*Daubert v. Merrell Dow Pharmaceuticals, Inc.* 1993). In practice, the criteria have operated very much like the checklist that Blackmun warned against. The second is the announcement of new interpretive rules, such as Judge Kozinski’s “litigation science” test, that rest on idealized, misleading, or misinformed assumptions about the scientific method. As a result, in attempting to diligently implement *Daubert*, federal judges have effectively fallen back on a “junk science” of how science works.

For those concerned with the quality of the science used in legal proceedings, it is important, then, to make sure that courts in the post-*Daubert* era rely on images of science that more accurately reflect what is known of the nature of scientific practice. This article contributes to that goal by reflecting on the role of representation in the sciences, both in and out of the litigation context. By seeing all scientific knowledge claims as representations, courts and analysts of the law will be better positioned to ask what makes some representations better or more reliable than others for purposes of legal fact-finding. The result may be a more nuanced application of *Daubert’*s core holding: that judges should be reluctant to admit evidence that satisfies no reasonable warrants of reliability. Understanding science as a special kind of representation should also provide defenses against untenable black-and-white rules that rest on untested judicial assumptions or, worse, on partisan assertions by experts. Adjudication, after all, is essentially a process of evaluating competing representations. Recognizing that science, too, only represents reality is a starting point for rethinking the judicial role in the assessment of litigation-generated science, and for realigning that role with the realities of judicial competence.

## Observing Science: Impartiality and Symmetry

Great gains in our understanding of science as a social process began in the 1970s with an “in effect” thought experiment that turned into a wide-ranging research program. Sociologists and anthropologists asked themselves how the making of science would look if one emptied one’s mind of prior conceptions of what science is or how it progresses. This meant divorcing the observation of science-in-the-making from any overarching theoretical definitions of the “scientific method,” such as Karl Popper’s view, endorsed by the *Daubert* court, that what distinguishes, or demarcates, science from nonscience is falsifiability (Popper 1959). [Citing Popper, the *Daubert* court observed, “Ordinarily, a key question to be answered in determining whether a theory or technique is scientific knowledge that will assist the trier of fact will be whether it can be (and has been) tested.”] The Popperian doctrine holds that it may not be possible to verify conclusively whether a given scientific theory is true, but that invalid theories can be conclusively falsified by empirical observations that conflict with the expected results. For courts confronted with the need to demarcate legitimate from illegitimate scientific claims in admissibility decisions, the falsifiability rule, and its associated practice of testing, seemed a godsend. After all, it is easy to ask whether a theoretical claim is testable or has been tested. But is falsification through testing how science actually operates, and can the criterion of falsifiability be meaningfully applied in practice? How could one answer questions such as these without observing in detail how working scientists conduct themselves? That is the observational challenge that professional students of science increasingly took upon themselves (Callon 1995).

David Bloor, a philosophically trained sociologist at the University of Edinburgh, set forth an influential programmatic approach to studies of scientific knowledge. Known as the “strong programme” in the sociology of scientific knowledge (SSK), that approach remains highly germane to present-day examinations of scientific validity in the legal context. Like Popper, Bloor and his successors in SSK were also concerned with the demarcation question that confronts judges in admissibility decisions: what makes an assertion about the nature of the world genuinely, or adequately, scientific rather than merely wishful, subjective, interested, grounded in superstition, or even deluded? To answer this question, the strong programme recommended an inductive and empirical approach, guided by four methodological principles:

- Causality: examine the conditions (psychological, social, and cultural) that bring about claims to a certain kind of knowledge.
- Impartiality: examine successful as well as unsuccessful knowledge claims.
- Symmetry: use the same types of explanations for successful and unsuccessful knowledge claims.
- Reflexivity: apply the same approach to sociology itself (i.e., to the investigator’s own claims to knowledge) (Bloor 1976).

Two of these principles, impartiality and symmetry, are of particular relevance in reconsidering how to interpret and implement *Daubert*. The impartiality principle in SSK presumes that much can be learned about how science works by looking at scientific claims that never established themselves as true. Specifically, one learns more about the scientific method by observing “science in action” (Latour 1987)—that is, how scientists sort out good from bad science in their actual practice—than by presuming to know in advance the constituents of that method (or those methods). The symmetry principle instructs the sociologist of scientific knowledge to be especially cautious about invoking nature (or, indeed, any type of explanatory variable, e.g., economic interest) one-sidedly, only to explain why a particular claim is true or false. After all, both sides in any scientific dispute need to contend with nature, and both are driven by multiple interests. The challenge for the SSK scholar is to determine why one side more than the other is deemed to have created the right representation of nature.

In part, the foundation for the symmetry rule is historical: during the course of a scientific dispute, the participants themselves do not know which side has the right answer. It is only after the conflict settles that truth becomes fixed in any sense, both for those involved and for future inquirers. Even then, scientific methods and findings are best seen as only provisional; what science “knows” at any given point is simply the best knowledge available to a particular community working in a particular paradigm, with particular assumptions, instruments, and techniques. In effect, then, the symmetry principle enjoins sociological inquirers not to think they know more about a dispute than scientists do themselves while that dispute is still in progress. Anything else would put the sociologist in the risky position of speaking from a higher plateau of knowledge than those engaged in producing the very knowledge whose success the sociologist is trying to understand.

The parallels to litigation should be clear. In litigation settings, as in the scientific controversies that SSK scholars study, we also encounter “science in action.” As in live scientific controversies, courts observe science in the making, because the facts needed to resolve legal disputes, and even the methods by which such facts might be ascertained, are seldom already out there in the existing scientific literature (Jasanoff 1995). Like the academic sociologist, the judicial fact finder is also interested in demarcating the claims that can be reliably relied on from those that are not sufficiently grounded in acceptable standards of scientific inquiry. To be sure, the law’s stake in demarcation is more explicitly normative and consequential: In the courtroom, reliable science serves as an aid to determining how disputes ought to be resolved, and hence to deciding who wins and who loses. And although historians and social scientists can afford to stand apart and observe scientists at work, judges cannot, because legal rules actually influence the production and testing of knowledge. But the potential intellectual traps are similar for both legal and sociological inquiry into the nature of science. The mistake, in law as in sociology, is to believe that there are pregiven, determinate standards, extrinsic to what scientists themselves would invoke when confronted by specific, competing claims. In effect, the best way to sort out the relative strength of expert claims in litigation is to adopt the SSK scholar’s impartial stance, and watch the opposing parties attempt to defend their positions. The ideal trial situation should in this respect imitate the ideal scientific laboratory.

From the standpoint of science studies, *Daubert* is flawed because it causes the judicial fact-finder to abandon both impartiality and symmetry, and to review challenged evidence without allowing it to engage in conversation with the challenger’s evidentiary claims. In the day-to-day practice of science, it is precisely the negotiation between competing viewpoints that discloses flaws in arguments and gives rise to preferred rules of method. An interesting parallel in the legal context can be found in the pre-*Daubert* history of the admissibility of DNA typing in U.S. courtrooms. There, the relative asymmetry of power and resources between the parties initially favored prosecutors, who successfully introduced DNA evidence, citing its near-infallibility, in hundreds of cases before they met substantial challenge in *People v. Castro* 1989. In that case, the first to deny admissibility, major flaws in the production of allegedly incontrovertible evidence were uncovered when expert witnesses for both sides staged an impromptu, mini-scientific controversy to resolve the issues between them. That debate, in turn, prompted a more systematic look at the foundations of DNA testing’s infallibility claims and led to methodological standardization and reform.

*Daubert*’s one-sided scrutiny sacrifices that kind of revealing dynamic in the interests of efficiency: Judicial denial of admissibility short-circuits potentially expensive trials. But what is the epistemological basis of the activist review that *Daubert* calls for? Unlike SSK researchers and other professional students of the scientific enterprise, judges are not subject to peer scrutiny or updating with respect to their mastery of what is, in the end, an extensive domain of philosophical, sociological, and political scholarship. Rather, *Daubert* and its progeny almost invite judges to invoke criteria that derive from their personal understandings, or misunderstandings, of how science works, thereby positioning the judiciary as virtually unreviewable SSK experts (Jasanoff 2001). *Daubert* accords to judicial folk-knowledge (i.e., cultural knowledge shared by judges) about the scientific process a privileged, almost insulated, position that it does not grant to the most eminent of expert witnesses.

All this is not to say that litigation is ever the ideal laboratory for testing scientific truth claims. Bias and distortion can indeed enter in many ways into the production of science for courtroom use, as they can into the production of science writ large (Krimsky 2003), and more aggressive judicial gatekeeping would be well warranted if it could serve as an effective filter against potential excesses of party-generated expertise. At the same time, judicial power should not be misused to prevent illuminating courtroom exchanges over scientific methods or to block the development of new information through litigation. How can *Daubert*’s legitimate interest in cleansing litigation of outrageous expert claims be balanced against the countervailing risk of letting judicial expertise function as its own form of unreviewable “junk science”? To find a way out of that dilemma, let us turn to the influential body of work that treats science as a mode of representation, specifically, as a set of strategies for representing nature.

## The Representational Turn

With increasingly rare exceptions, scientists today can neither see the natural phenomena they claim to be investigating nor directly show them to others. No human eye has seen climate change or biodiversity loss; an oncogene, the human genome, or the complete array of chemical elements; schizophrenia or hypertension; the ozone hole, the eye of a hurricane, or the AIDS virus. Some of these, such as the ozone hole, the eye of Hurricane Katrina, or the virus that causes AIDS, are visually familiar to us through complex magnification, photographic, and coloration techniques. Medical conditions, such as hypertension or schizophrenia, are made palpable through techniques of measurement or expert diagnosis that redescribe the pathology in standardized terms, including numerical scales and specialist language. Many scientifically known objects, such as the human genome or the array of chemicals, are rendered “visible” only through translation into readable visual forms: the familiar ACTG alphabet of DNA’s base pairs, or the classic periodic table of the elements. Still others, such as climate change and biodiversity loss, are known only through charts and graphs that convey some pieces of a more complex whole, such as the now-famous readings of seasonal rises and falls in atmospheric carbon dioxide concentrations at the Mauna Loa observatory in Hawaii.

Science, as Bruno Latour most clearly demonstrated in his influential early work (Latour 1990), can usefully be thought of as a conglomerate of inscriptions, or visual records, which make knowledge portable across spatially and culturally unconnected domains. When representations are successful, in the sense that no one any longer contests their basic meaning, they become in Latour’s words “immutable mobiles” (Latour 1990). They move across time and space without constantly raising new questions about what they are or what they mean. Of course, it may take decades, even centuries, for bits of science to become immutable in this way. Throughout the process of creating stable representations (or, perhaps more accurately, stable techniques of representation), conflicts abound: over the adequacy of models and the accuracy of measurements, the right ways of reading inscriptions, and the wider meanings that can be extrapolated from such records, taken alone or brought into interaction with one another. For many years, science studies research has documented in microscopic detail the kinds of work needed to convert these sorts of natural objects and phenomena into stable forms that can be seen, manipulated, and used for scientific communication—in contexts ranging from grant applications to expert evidence. But eventually, representations become standard, at least within specific domains of practice. Ken Alder’s account of a seven-year controversy in revolutionary France to standardize the meter is an example (Alder 2002). No one any longer seriously questions the length of the meter in ordinary use, any more than anyone contests the biological meaning of sperm or stegosaurus, systolic blood pressure, or sickle cell anemia.

In the contemporary world, the law is very often implicated in stabilizing scientific representations. Legal proceedings serve in effect as “agonistic fields” (Latour and Woolgar 1986) or fields of contestation, in which experts debate the merits of competing representations and the techniques that produced them. New knowledge often emerges as a result of this process, both about the nature of disputed phenomena and about the cause–effect relationships of concern to the law. Medical diagnostic criteria for syndromes such as posttraumatic stress disorder, for example, arose hand in hand with the efforts of sufferers to gain compensation for such conditions or to use them in criminal defense (Young 1995). Much that we know today about the toxicity of such chemicals as dioxin or the long-term health effects of radiation, asbestos, and methyl isocyanate (the gas released from a Union Carbide plant in Bhopal, India, in 1984) was learned in the form of evidence generated by plaintiffs’ experts in tort litigation. The conclusion that silicone gel breast implants do not cause immune system disorders was reached after more than 20 years of litigation-driven science (Saul 2006). Forensic sciences form an entire subfield of technical knowledge that owes its very existence, not to mention its objects, instruments, and methods of analysis, to the knowledge-generating interplay of science and the law.

The history of forensic DNA typing illustrates these dynamics especially well. A DNA fingerprint is a scientific inscription of special utility in law enforcement: It represents a person’s identity as a configuration of parallel lines resembling a somewhat smudgy supermarket bar graph, with bands of varying thickness corresponding to the presence of particular alleles. These fingerprints are deemed virtually unique, because the chances that the same allelic pattern will be found in two people who are not identical twins are vanishingly small. When DNA fingerprints were first introduced as evidence in the 1980s, prosecutors used them not only to match a suspect’s DNA to DNA traces found at the crime scene, but also to support estimates of the probability or, more properly, improbability of an accidental match or false positive. Dizzyingly low numbers, coupled with unshakeable expert confidence about the validity of the matches, at first concealed the tacit judgments that were inevitably involved in reading the so-called fingerprints. For example, experts sometimes declared matches between crime scene samples and suspect samples without explaining why they had ignored the presence of an extra bar in one fingerprint, or the systematic displacement of bars between two allegedly identical fingerprints. It also emerged that probabilistic estimates initially had not taken into account the possibility of reduced allelic variation within ethnic subgroups, a factor that increases the likelihood of false positives. *People v. Castro* 1989, discussed above, helped bring these issues into the open. It took years of legal contestation, two expert committee reports from the National Academy of Sciences (National Research Council 1992, 1996), and extensive standardization by the Federal Bureau of Investigation and state crime laboratories to achieve a near-uniform national standard of good practice for forensic DNA testing. Judged by this standard, the practices used to convict defendants in the early cases clearly fell short, but it took an evolutionary, law-driven process to reveal and correct the most egregious flaws.

Another context in which litigation has helped to push forward a particular form of scientific representation is brain imaging (Dumit 2003). In this case, the pressure to use a new technology originated partly in the criminal defense community. Lawyers sought to establish with the aid of brain scans that their clients were acting in a state of diminished mental capacity. A noted case was that of John W. Hinckley Jr., the man who in 1981 shot President Ronald Reagan and three other people in order to impress the actress Jodie Foster, with whom he had become obsessed through her role in *Taxi Driver* (Dumit 2003). In *Roper v. Simmons* 2005, evidence from brain scans was introduced by scientific and professional *amici curiae*, including the American Medical Association and the American Psychiatric Association (AMA, APA et al. 2004), to establish that adolescent brains do not function at the same levels as those of adults. These submissions played a part in persuading the Supreme Court to declare the death penalty unconstitutional for persons younger than age 18, although some have argued that the neuroscientific evidence offered to the Court was itself immature and should not have been advanced as a basis for decisions of constitutional significance (Schaffer 2004).

Both DNA fingerprints and brain images prominently involve visual representations of biological facts, but scientific representations can take many other forms, not all pictorial or graphic. Another particularly persuasive mode of representation is statistical. The power of statistics derives from aggregation, which allows signals to be detected that might have escaped notice if perceived only as random events (as in the case of cancer caused by diethylstilbestrol, or DES), and from regression, which allows putative cause–effect relationships to be either demonstrated (as in the case of passive smoking) or discredited (as in the case of Bendectin) by comparing large numbers of cases. So important has statistical evidence become in litigation that the Federal Judicial Center includes a chapter on that topic in its *Reference Manual on Scientific Evidence* 2000. As in other areas of litigation science, however, appropriate statistical methods often do not preexist the dispute in question, but develop only as parties in a controversy actively sort out which end points, which causal factors, and which populations should be subjected to statistical investigation in the first place. Of course, such activities may fail to support claims brought forward by the plaintiffs’ experts, but by shining a brighter light on the evidence, and by sometimes triggering reanalyses of older data, they may also reveal methodological flaws and previously unsuspected correlations. An example is the dispute over the increased risk of suicidality (suicidal thinking and behavior) in children and adolescents caused by antidepressant medications that were once considered safe for use. On 15 October 2004, following years of litigation and complaints by victims’ families, the U.S. Food and Drug Administration issued a public health advisory on these drugs on the basis of a meta-analysis of 24 studies including 4,400 patients (U.S. Food and Drug Administration 2004).

## Forensic Representation: The Rules of the Game

Science, we have seen, necessarily involves argument and representation in order to be persuasive. Yet, as all agree, the representation of science in the courtroom occurs under rules that are crucially different from those of the scientific workplace. Critics of the legal system frequently fall into the trap of asymmetry in characterizing those differences. They assume that representation within the sciences is neutral, impartial, and objective; by contrast, legal representation is seen as deviant (“junk science”), because it incorporates such distorting factors as the interests of parties, their experts, and their legal counsel. Or, like Judge Kozinski, critics see science as governed by a monolithic set of methods and practices, such as universal standards of transparency and peer review; by comparison, “litigation science” seems to fall short.

Such oversimplified analysis not only misrepresents the nature of litigation science but also endangers the productive use of large amounts of science generated in the course of legal proceedings. All scientific claims-making, after all, is driven by interests of varying kinds—intellectual, economic, institutional, and cultural. Indeed, much science produced to serve the needs of public policy, particularly in areas of health, safety, and environmental regulation, is generated by private actors who have substantial stakes in the outcomes of the policy process. Moreover, a great deal of published science passes through only the most superficial peer scrutiny and is never tested, replicated, or even cited. Scientific fraud and misconduct are well-known indicators of those realities (Broad and Wade 1985).

Rather than contrasting litigation science with a nonexistent ideal of research science, a more fruitful approach to the law’s demarcation problem is to ask what is special about the forensic representation of science, and how that stylized form of representation differs from conventional modes of scientific representation. For that inquiry, it is useful to think of litigation science not as an intellectually different kind of enterprise from research science, but rather as a performance subject to distinctive rules of the game. Empirically, we can ask in what respects the courtroom functions differently from the laboratory or the scientific journal as a theater for staging scientific representations. That move allows us to focus on the actual strategies of presentation and representation used in litigation. It enables us to see that scientific representation is not merely the product of more or less well-intentioned experts submitting to more or less effective cross-examination before more or less competent juries. Looking at evidence-giving as a kind of performance sheds light on the critically important roles of judges and lawyers in configuring—or framing—the manner in which evidence is presented. It provides a bridge to delineating more precisely the difference between forensic and other forms of scientific representation.

Judges play a crucial role in framing the presentation of expert evidence throughout a trial, far beyond the conduct of the admissibility screening. As custodians of order and routine in the courtroom, judges can influence not only what but how evidence is brought forward, and who interprets it for the jury. Thus, in the 1982 Hinckley trial, the presiding judge, Barrington D. Parker, reluctant to overpower the jury with visual evidence of the defendant’s alleged insanity, at first rejected the brain scans introduced by the defense. Later, after he admitted the scans as relevant, Parker directed them to be projected so far away and on such a small screen that the message was quite possibly lost in translation (Dumit 2003). In the 1995 murder trial of O.J. Simpson, Judge Lance Ito determined that the DNA test protocol used by Cellmark Laboratories, the laboratory used by the prosecution, was appropriate and did not need to be offset by other techniques selected by the defense. He accordingly denied a defense request to split the available blood samples to permit independent testing to be conducted (Jasanoff 1998b). Ito also excluded expert testimony seeking to interpret video evidence for the jury, arguing that any viewer could make sense of such testimony unaided by experts, because it appeals directly to people’s communal sense of sight. The judge treated the videotape as a self-contained, objective record needing no further explication by expert witnesses.

But video testimony has been interpreted by experts in other cases, and those interpretations have sometimes proved extremely consequential. The 1992 and 1993 trials of Los Angeles police officers in the beating of Rodney King provide a telling example. In the first trial, the prosecution presented a videotape shot by a chance bystander showing King being brutally beaten by a group of policemen; like Judge Ito in the Simpson trial, the prosecutors thought the tape spoke for itself and needed no further commentary. Defense lawyers, however, called upon Sergeant Charles Duke of the Los Angeles Police Department (LAPD) to interpret the video as an expert on the use of force. Duke’s testimony in effect translated into the language of professional judgment what looked to the untutored eye to be an extremely violent beating of one man by several others. Within the context of police practice, Duke argued, it was King’s bodily movements that were aggressive, and the beating was the legitimate response of officers trained to respond forcefully to such acts of aggression (Goodwin 1994). Interviewed on Court TV, a defense lawyer said that the object had been to show that “[w]hat looks like uncontrolled . . . brutality and random violence is indeed a very disciplined and controlled effort to take Mr. King into custody” (Goodwin 1994). The attempt to professionalize the reading of the video was successful, and all four LAPD officers charged with using excessive force were acquitted. In a federal trial a year later, two of the same officers were found guilty of having violated Rodney King’s civil rights, on the basis of the same visual evidence.

**Figure 1:**
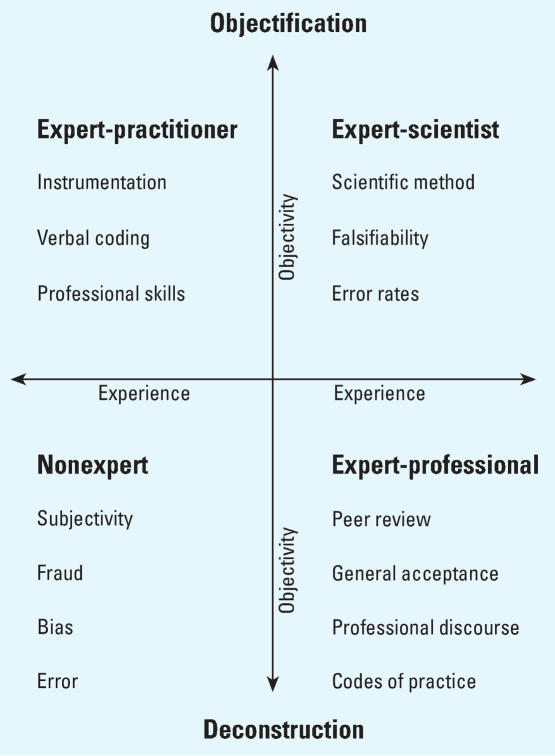
The game board of expertise.

These episodes illustrate the importance of the courtroom as a performative space in which strategies for excluding or mobilizing expertise can change the very way juries perceive the evidence. Of course, such performances are by no means restricted to legal settings. In science as well as in law, experts are relied on to reduce ambiguity, to make it appear as if only one story can be told on the basis of the available evidence. For this purpose, in science as in the law, experts must pattern as impartial and objective truth-tellers: in the agonistic fields of science as of law, the persuasiveness of an argument depends on garnering maximum credibility for it, while sowing doubt and uncertainty about any alternative interpretations. This dynamic plays out on what I have called the “game board of expertise” (Jasanoff 1998a). The game is symbolically enacted on a board defined by two axes labeled, respectively, experience and objectivity (Figure 1). The aim is to position one’s own claims of expertise as high as possible on both axes, while seeking to demote the opponent’s claims.

On this game board, various moves can be used to move experts away from the nonexpert quadrant, defined as least experienced and least objective, toward the quadrant of the expert-scientist, defined as most experienced and most objective—or vice versa. Some of these strategies are widely used in both scientific and legal settings, although under different constraints and in relation to different audiences. Thus, to maximize credibility along the experience axis, an expert can be positioned as a mainstream member of a recognized professional group, well versed in that group’s discourses and codes of practice; in legal terms, this strategy is similar to meeting the “general acceptance” test articulated in *Frye v. United States* 1923 and reiterated in *Daubert*. Along the objectivity axis, credibility-enhancing moves may include meeting tests of the kind articulated in *Daubert* that help to demonstrate the scientific validity of the expert’s approach to generating new knowledge. Although the focus on this axis is on method rather than experience, the goal, again, is to show that the methods themselves are not ad hoc or case specific but are recognized as valid and credible by a body of peers.

In science as in law, claims to both experiential and scientific expertise can be negated through allegations of subjectivity, bias, fraud, and error. In neither context can one expect peer judgments of competence or validity to be consistent or foolproof. The results of particular credibility-building processes will depend on who is deemed to be a peer and how carefully such persons exercise their critical faculties. In short, even within the sciences, there is no scientific means of assessing the credibility of expert representations.

Perhaps the most striking difference between the dynamics of establishing expert credibility in law and in science flows from the factually specific and ad hoc character of many legal proceedings. Unlike “normal science,” which by definition operates within the constraints of a paradigm, or set of communally recognized rules and practices, the technical questions generated by the law often seem to come out of nowhere, and sometimes to go nowhere, in the sense of providing starting points for new scientific inquiry. It is in these one-off or stand-alone cases that the law’s technical fact-finding capacity is at its most vulnerable, because expertise then is constructed entirely within the four corners of the legal process. Sergeant Duke’s role in the first Rodney King trial graphically illustrates this point. Even this nonscientist practitioner could be made to look expert, as a person possessing special skills in diagnosing and analyzing a particular kind of problem (in this case, the use of excessive force by the police). In a context such as this, there is no external world of practice that can be referred to for validation; the law can only look inward upon itself as it attempts to determine what counts as relevant expertise and who counts as an authoritative expert.

## Drawing Lessons: Rethinking Demarcation

It follows from all this that the model of judge-made demarcation proposed in *Daubert* and further elaborated by Judge Kozinski in *Daubert II* (*Daubert v. Merrell Dow Pharmaceuticals, Inc.* 1995) is seriously defective. Both decisions buy into the faulty premise that there are exogenous criteria, lying outside the legal process, by which judges can distinguish between good and bad science. Both uncritically assume that judges will be able to ascertain these criteria and objectively apply them to challenged evidence without allowing controversies to unfold and methodological disputes be sorted out in the process. Neither takes into account the dynamics of litigation itself, including gaming by the parties and framing by judges, as constitutive factors in the production and representation of knowledge, so that admissibility operates as only the first step in the process of re-representing scientific knowledge for courtroom use; what is admitted, in other words, is not precisely what a jury sees, as became clear in the Hinckley trial. And neither opinion is informed by the wealth of existing scholarship on the production and validation of scientific facts. An unreflective application of the *Daubert* criteria thus puts courts at risk of producing and applying a “junk science” of the nature of scientific knowledge.

How could courts do better? One approach consistent with the symmetry principle in the strong programme of the sociology of scientific knowledge would be to let the parties themselves do more of the work of demarcation, with judges acting as referees between the parties rather than as custodians and enforcers of transcendental standards of good scientific practice. Litigation science, after all, is a particular form of science-in-the-making. If the criteria for generating valid and relevant science are not available in advance, but need to be worked out in the very process of establishing case-specific facts, then courts might seek to promote good practices by intervening earlier in that process, through well-designed pretrial agreements. Judicial power clearly extends to orchestrating the relevant conversations between or among parties to a science-intensive controversy, thereby brokering the design of improved methods and protocols to fill in gaps in the evidence. Among the issues that parties might be asked to resolve in pretrial proceedings are the following:

- What new or additional information is needed?
- What needs to be done to obtain it; for example, what protocols and standards of proof are appropriate?
- How study results should be reviewed?
- What should be done with suggestive but inconclusive evidence?

Judicial involvement in such negotiations could include the safeguarding of certain traditional concerns for fairness that were overlooked in *Daubert*’s “thinking like a scientist” mandate. For example, in pretrial discussions, judges could seek actively to ensure that all parties with interests in the proceedings are represented and have a chance to bring their expertise to the bargaining table. The issue of costs could be explicitly addressed, and courts could impose equitable rules for sharing the cost of developing new evidence—taking into account the reasons why such knowledge was not available from the outset. Similarly, procedures for peer reviewing new studies could be worked out under judicial supervision, and agreements could be made about how to reach closure in the event that studies prove inconclusive. None of this would be easy, and adding such processes at the front end of litigation might entail very considerable expense. At the same time, such pretrial activity might reduce the ultimate cost of litigation, while improving the quality of science generated through the adversary process.

Judicial refereeing of litigation science-in-the-making would have the further advantage of acknowledging that lawsuits today serve less as testing grounds for competing claims than as devices for prompting the discovery, production, and assessment of new knowledge. As the cost of litigation spirals, trials play a less and less prominent role in the workings of American law (Galanter 2004). This means that the greatest part of dispute resolution, including most debate over the quality and sufficiency of scientific evidence, occurs outside the framework of the trial; yet, the stylized drama of trial advocacy and its potential for distortion continue to dominate the thinking of most observers and critics of litigation science, including Justice Blackmun in *Daubert*. That error of emphasis could be avoided by shifting attention away from admissibility decisions, which figure in only a small fraction of lawsuits, to a more balanced and symmetrical consideration of the strengths and weaknesses of the available evidence.

## Opening the Gates to Litigation Science—Judiciously

In retrospect, *Daubert*’s gatekeeping metaphor appears to have misconceived both the timing and the appropriate nature of judicial intervention into the production of litigation-related scientific knowledge. *Daubert* and its progeny conceived of scientific and technical expert testimony almost as an assault on the courtroom: the innermost citadel of the law. Like zealous custodians barricading the fortress gates against barbarian invaders, judges in the post-*Daubert* era were empowered to shut the courtroom gates to expert testimony that they deemed irrelevant or unreliable. In carrying out their mandate from the Supreme Court, federal judges found themselves in the unenviable position of serving in effect as final, largely unreviewable experts on what constitutes “the scientific method.”

By looking at science as a form of persuasive representation, and by importing the ideas of impartiality and symmetry from the sociology of scientific knowledge, we can radically reconceptualize the judicial role in relation to scientific evidence: from gatekeeping to refereeing. As referees of science-in-the-making, judges would focus on the process through which litigation science is generated rather than on its validity or invalidity. They would be in a position to structure agreements among the parties that would be most conducive to producing relevant and reliable knowledge. With an eye on the dynamics of knowledge production, particularly on the game board of expertise, judges could allow the disputing parties themselves to identify and resolve their epistemological differences in an orderly fashion. Not least, judicial refereeing might ensure that the costs of producing missing information and the burdens of uncertainty would be equitably distributed.
